# Portal Vein Thrombosis and Intra-Abdominal Hypertension Presenting as Complications of Hypertriglyceridemia-Induced Severe Acute Pancreatitis

**DOI:** 10.7759/cureus.9889

**Published:** 2020-08-20

**Authors:** Afshin Amini, Zahra Vaezi, Elliott Koury, Sajid Zafar, Elie Chahla

**Affiliations:** 1 Internal Medicine, St. Luke's Hospital, Chesterfield, USA; 2 Internal Medicine, Zahedan University of Medical Sciences, Zahedan, IRN; 3 Gastroenterology and Hepatology, St. Luke's Hospital, Chesterfield, USA

**Keywords:** portal vein thrombosis, intraabdominal hypertension, hypertriglyceridemia-induced acute pancreatitis

## Abstract

A 44-year-old male without any significant past medical history presented to the emergency department (ED) with the chief complaint of severe constant epigastric pain for three hours. On physical examination, the abdomen was distended and tender, particularly in the epigastric region. The lab work showed an elevation of the lipase (12,405 U/L) and triglycerides (5,837 mg/dL). An abdominal CT scan with contrast was ordered, which revealed non-necrotic pancreatitis. In addition, the liver ultrasound showed no evidence of gallstones. Subsequently, fluid infusion, meropenem, pain medication, and an insulin drip were started, and the patient was transferred to the intensive care unit (ICU). After six hours in the ICU, he complained of abdominal pain despite taking a high hydromorphone dose.

On further physical examination, the abdomen was tender and distended but without rebound tenderness. The gastric distention on kidneys, ureter, and bladder (KUB) and a bladder pressure of 34 mmHg raised the suspicion for intra-abdominal hypertension (IAH), which led us to place a nasogastric tube (NGT) and consult the surgical team. The patient's symptoms and bladder pressure were closely followed and showed significant improvement. On day seven in the ICU, the patient responded well to medications; feeding through the Dobhoff tube was started, and his triglycerides decreased to approximately 1,000 mg/dL. Despite his general improvement and meropenem regimen, the patient spiked a fever of 38.5 °C. Due to the possibility of pancreatitis complications, a CT abdomen with contrast was ordered, which showed partial portal vein thrombosis (PVT). Subsequently, enoxaparin was started, and the patient was closely observed for gastrointestinal bleeding. Eventually, after 17 days in the ICU, the patient was transferred to the floor and then discharged from the hospital with normal lab tests and without evidence of portal thrombosis on abdominal CT.

In this report, we illustrate and discuss a case of hypertriglyceridemia (HTG)-induced pancreatitis (HTGP), which progressed to PVT and IAH. Physicians should be aware that patients with HTG are inclined to have severe pancreatitis. In addition, the degree of triglyceride elevation is correlated with the severity of acute pancreatitis.

## Introduction

Acute pancreatitis is diagnosed when two of the following three criteria are met in a patient: acute severe onset epigastric pain usually radiating to the back, serum lipase or amylase level elevation to three times or more than the upper limit of normal, and characteristic signs of pancreatitis on radiography (contrast-enhanced CT, MRI, or transabdominal ultrasonography) [1]. Hypertriglyceridemia (HTG) is considered a significant risk for acute pancreatitis when levels are greater than 1,000 mg/dL (11.3 mmol/L) [2]. Triglycerides themselves do not appear to be toxic; however, their breakdown products may be. Triglycerides are broken down into toxic free fatty acids (FFA) by pancreatic lipases and these may be a cause of lipotoxicity during acute pancreatitis [3].

The risk of developing HTG-induced pancreatitis (HTGP) increases with higher triglyceride levels. The risk of HTGP with serum triglycerides of >1,000 mg/dL (11.3 mmol/L) is approximately 5% in comparison to 10-20% with triglyceride levels of >2,000 mg/dL (22.6 mmol/L) [4]. In addition, the degree of triglyceride elevation is associated with the severity of acute pancreatitis [5]. Furthermore, patients with HTGP tend to have more severe pancreatitis as compared with patients with other causes of pancreatitis [6]. HTGP makes up 1-14% of all cases of acute pancreatitis and up to 56% of pancreatitis cases during pregnancy [7]. In a prospective study involving 400 acute pancreatitis cases by Nawaz et al., HTGP was noted to be more common in young diabetic obese male patients [8].

In this report, we present a case of HTG-induced severe acute pancreatitis (SAP) that progressed to portal vein thrombosis (PVT) and intra-abdominal hypertension (IAH) during the course of admission.

## Case presentation

**Figure 1:**
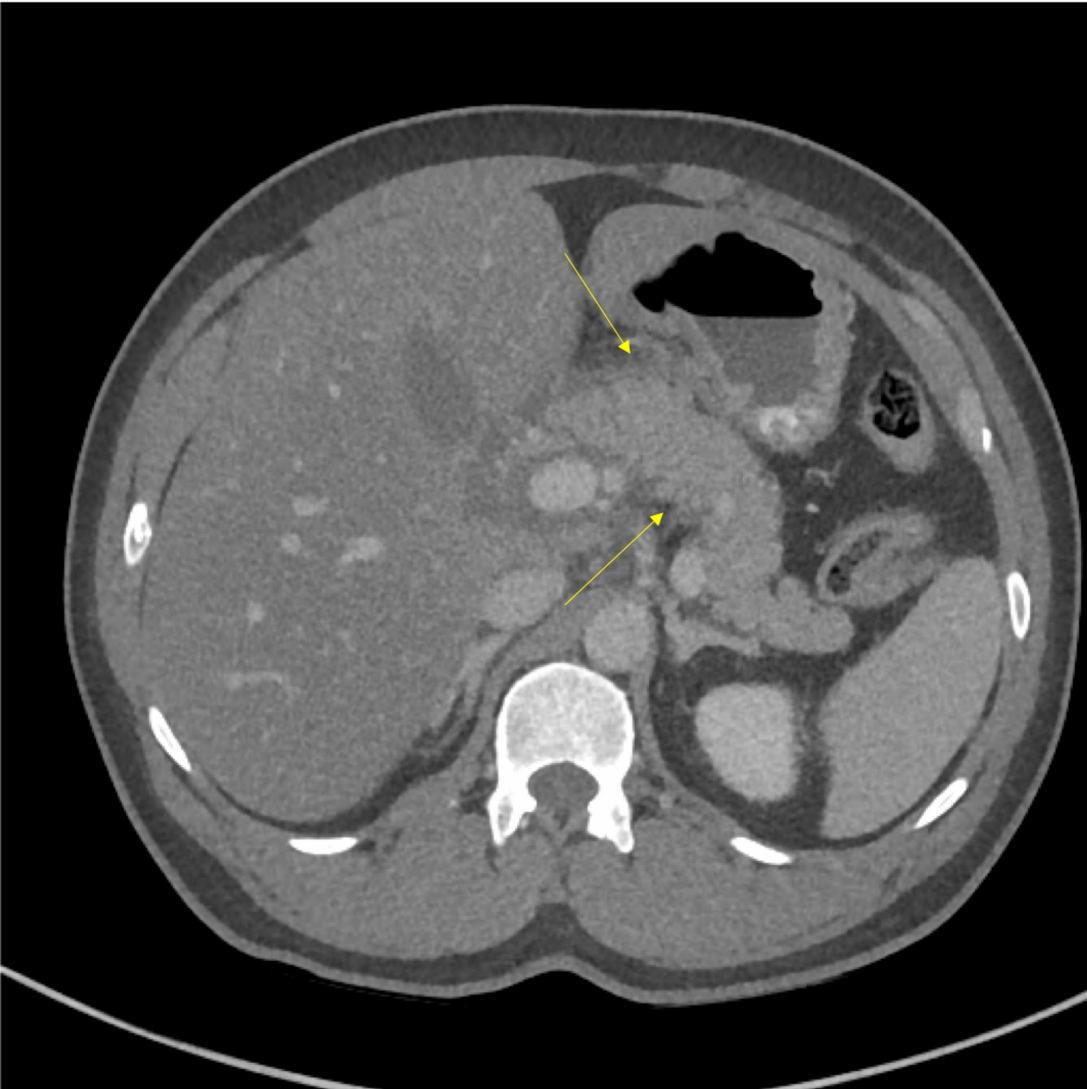
Abdominal CT scan with contrast The image shows non-necrotic pancreatitis (yellow arrows) CT: computed tomography

The patient was a 44-year-old male without any significant past medical history who presented to the emergency department (ED) with complaints of severe 10/10 epigastric pain for approximately four hours. The patient described the pain as sharp, stabbing, epigastric abdominal pain that radiated to the back. The examination was notable for generalized abdominal tenderness mostly on the epigastric region without any masses or noted rebound. The patient did not have a history of smoking or alcohol consumption and took no home medications. Initial laboratory workup was significant for white blood cell (WBC) count of 19.2 K/uL (normal range: 4.3-10 K/uL), hematocrit of 50% (normal range: 40-48%), potassium of 5.4 mmol/L (normal range: 3.5-4.9 mmol/L), glucose of 269 mg/dL (normal range: 65-110 mg/dL), hemoglobin A1c of 8.3% (normal range: 0-6%), lipase of 12,405 U/L (normal range: 23-300 U/L), C-reactive protein (CRP) of 18.7 mg/dL (normal range: 0-9 mg/dL), cholesterol of 452 mg/dL (normal range: ≤199 mg/dL), triglyceride of 5,837 mg/dL (normal range ≤149 mg/dL), lactate dehydrogenase (LDH) of 412 U/L (normal range: 140-280 U/L), calcium of 5.9 mg/dL (normal range: 8.2-10.4 mg/dL), total bilirubin of 6.9 mg/dL (normal range: 0.2-1.3 mg/dL), and a calculated Ranson’s score of 8, indicating severe pancreatitis. An abdominal CT scan with contrast revealed non-necrotic pancreatitis with an association of duodenitis (Figure 1).

**Figure 2:**
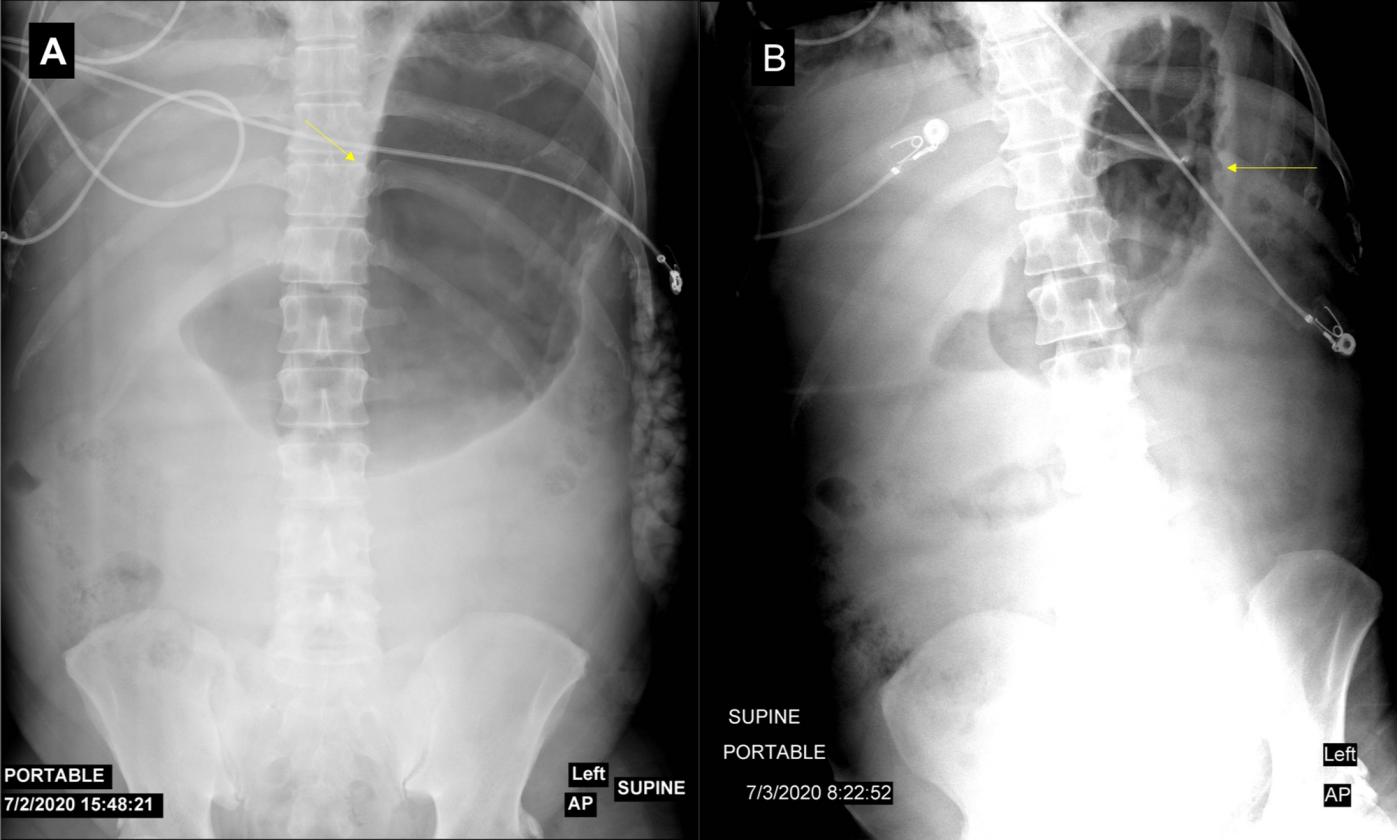
Kidney, ureter, and bladder (KUB) X-ray A: the image shows gastric distention (yellow arrow). B: the image shows gastric decompression after the insertion of the nasogastric tube (yellow arrow)

An ultrasound of the gallbladder was done upon admission, which showed no evidence of biliary stones. The patient was kept nil per os (NPO) and was started on 250 mL/h of lactated Ringer’s solution. The pain was controlled with intravenous hydromorphone, and the patient was transferred to the ICU for close monitoring. After six hours, the abdominal pain worsened, and the patient developed abdominal distension and increased bladder pressure (34 mmHg). A kidney, ureter, and bladder (KUB) X-ray revealed significant gastric distention (Figure 2A). Given the suspicion of abdominal compartment syndrome (ACS), the patient was placed in a supine position and received muscle relaxants. A nasogastric tube (NGT) was inserted and the surgical team was consulted. The patient was managed with these conservative measures, and a follow-up KUB (Figure 2B), bladder pressure, and abdominal examination showed significant abdominal decompression.

**Figure 3:**
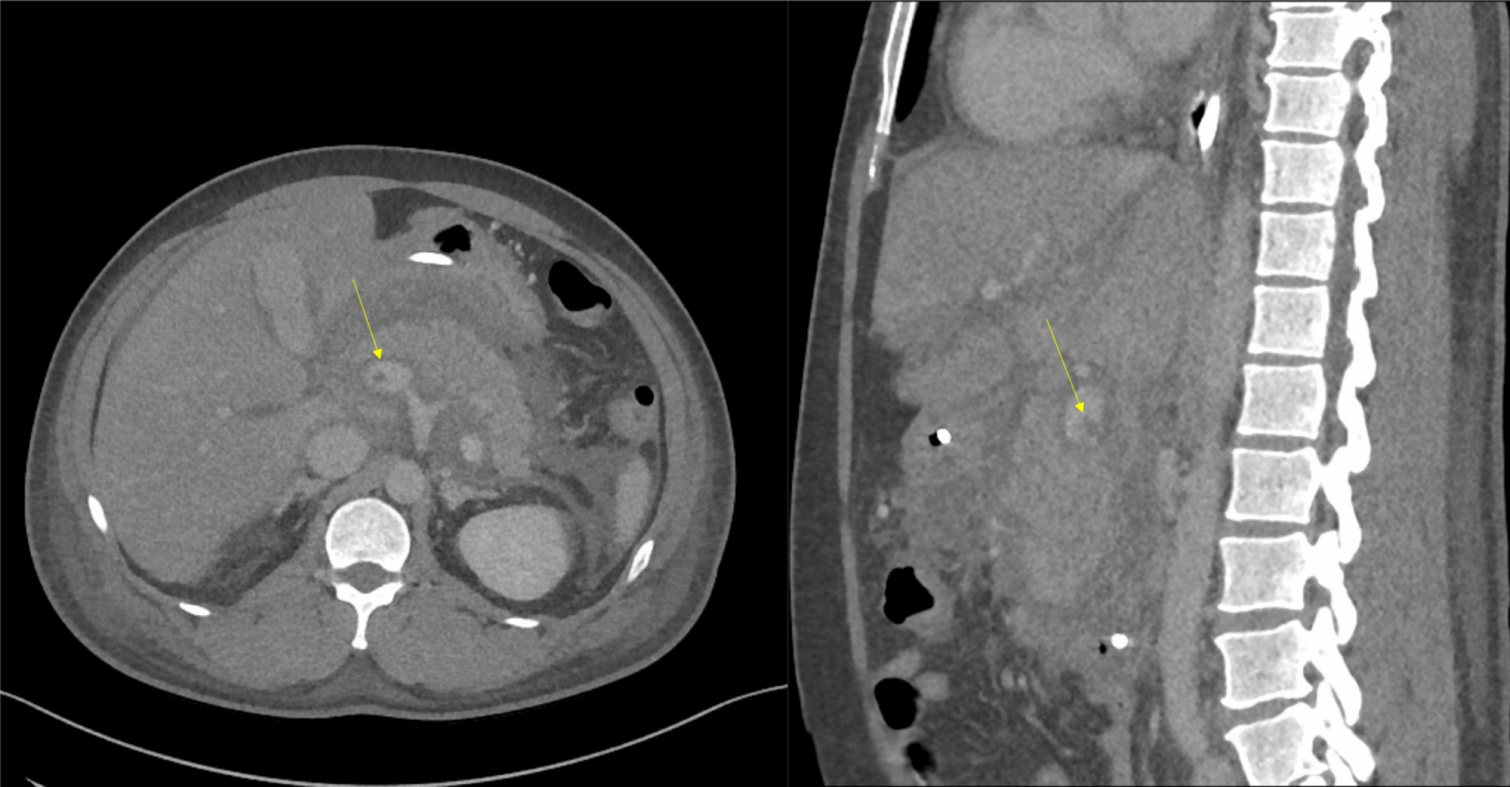
Repeat abdominal CT scan with contrast The image shows partial portal vein thrombosis (yellow arrows) CT: computed tomography

After five days, feeding through a Dobhoff tube was initiated. Despite the improvements in abdominal tenderness and triglyceride levels, one episode of fever (38.5 °C) was observed. A repeat CT scan of the abdomen with contrast revealed partial PVT (Figure 3), and enoxaparin was initiated.

After 17 days in the ICU, the patient was transferred to the floor and eventually discharged from the hospital on fenofibrate, metformin, and apixaban. At discharge, his lab tests, including complete blood count (CBC), comprehensive metabolic panel (CMP), and triglyceride levels, were within normal limits, and abdominal CT with contrast showed complete resolution of PVT. The plan after discharge was to repeat abdominal CT in six weeks and continue apixaban for six months.

## Discussion

PVT and abdominal hypertension were two complications of HTG-induced acute pancreatitis that were seen in our patient. Pancreatitis can cause a range of venous and arterial vascular complications. Major vascular complications of pancreatitis occur with a frequency of 1.2-14%, with a greater incidence seen in chronic pancreatitis (7-10%) than in acute pancreatitis (1-6%). Of these complications, venous thromboses are more commonly reported in the splenic vein and less frequently in the portal vein or superior mesenteric vein [9]. According to the location of splanchnic vein thrombosis (SVT), the pooled prevalence of portal vein, splenic vein, and mesenteric vein thrombosis in pancreatitis is 6.2%, 11.2%, and 2.7% respectively [10]. Thrombotic complications, as reported in the literature, are more commonly associated with alcohol-induced, necrotizing, and chronic pancreatitis, as opposed to acute pancreatitis [11]. Portosplenomesenteric venous thrombosis (PSMVT) develops in approximately 50% of patients with necrotizing acute pancreatitis and is rare in the absence of necrosis [12]. The pathogenesis of venous thromboses can be explained by stasis, spasm, and mass effect resulting from the surrounding inflamed pancreas and direct damage to the venous wall by released enzymes [13].

In a retrospective study by Mortele et al. on 100 patients with acute pancreatitis, the presence of splenic and superior mesenteric vein thrombosis was significantly correlated with the severity of pancreatitis. However, thrombosis of the portal vein was not correlated with pancreatitis severity. This finding is important since it indicates that clinicians and radiologists should be aware of this complication even in patients with “moderate” pancreatitis [14]. Acute PVT is often clinically silent and may be diagnosed during radiologic evaluation for other reasons, such as acute pancreatitis. Other patients may present with abdominal pain that develops suddenly or progresses over a few days. Patients may also report fever and dyspeptic symptoms [15]. The diagnosis of PVT in the setting of acute pancreatitis can be challenging due to abdominal pain being attributable to either PVT or to pancreatitis itself. In our case, the presence of fever despite the use of broad-spectrum antibiotics was the manifestation of PVT. Acute PVT is managed with anticoagulation to prevent extension of the clot and to allow for recanalization so that intestinal infarction and portal hypertension do not develop. Treatment is usually recommended for three to six months [16].

The incidence of IAH in patients with SAP is approximately 60-80%. It is usually an early phenomenon and partly related to the effects of the inflammatory process, which can lead to the development of retroperitoneal edema, fluid collections, ascites, and ileus. This can also be partly iatrogenic, resulting from aggressive fluid resuscitation [17]. IAH has been defined as an intra-abdominal pressure (IAP) of 12 mmHg or greater [18]. For the measurement of abdominal pressure, 25 ml of sterile saline is instilled in the urinary bladder. Subsequently, the hydrostatic pressure is measured in mmHg with IAH being classified into grade I (12-15 mmHg), grade II (16-20 mmHg), grade III (21-25 mmHg), and grade IV (>25 mmHg) [19]. IAH and ACS are typically early phenomena in SAP. According to most reports, IAH develops in the first three to five days after hospital admission [20].

As ileus and gastroparesis are often present in acute pancreatitis, reducing the intraluminal volume of the gastrointestinal tract is a logical first step. In the case of gastric dilatation, nasogastric decompression can easily be done and may have a significant impact on IAP [20]. Our case, with severe IAH, showed an excellent response to the insertion of NGT and the use of muscle relaxants.

## Conclusions

Pancreatitis in the setting of severe HTG is a unique and often underdiagnosed disease with significant morbidity and mortality rates. When suspected, prompt diagnosis and treatment should be initiated. Supportive therapy should be initiated to treat acute pancreatitis, glucose levels need to be controlled, and subsequently, triglyceride levels need to be reduced with the aid of lifestyle modifications and pharmaceutical agents. Additionally, physicians should be aware of the potential complications of pancreatitis such as venous thrombosis and IAH, even in the setting of non-necrotic pancreatitis, as was presented in our case.
